# The use of problem-specific and strategic treatment add-ons in inpatient psychiatric care

**DOI:** 10.1017/S003329172510086X

**Published:** 2025-06-24

**Authors:** M. David Rudd

**Affiliations:** Department of Psychology, University of Memphis, Memphis, TN, USA

**Keywords:** evidence-based psychological treatment, inpatient hospitalization, suicide, problem-specific treatment add-ons, elevated suicide risk

Hawes, Marcello, and Kleiman (2025) offer an excellent review of the unique challenges facing inpatient psychiatric care and the scarcity of relevant clinical research and provide a much-needed call to action to fill this critical gap in the literature. As they rightly note, the limited use of evidence-based psychological treatments (EBTs) in the inpatient setting are the consequence of a convergence of factors, with the average length of stay of only 3–7 days in the U.S. being a formidable challenge in efforts to adapt and use EBTs developed for much longer treatment timeframes in other clinical settings. As Ward-Ciesielski and Rizvi (2021) suggest, this gap in the use of EBTs in inpatient settings has the potential for iatrogenic effects, particularly for those hospitalized secondary to elevated suicide risk, which is one of the most common reasons for admission.

Hawes et al. (2025) cited our recent inpatient randomized clinical trial (RCT) comparing treatment as usual (TAU) plus an adaptation of Brief Cognitive Behavioral Therapy for Suicide Prevention (BCBT-I) to TAU alone (Diefenbach et al., 2024). It is important to mention that TAU did include isolated suicide prevention measures routinely used in inpatient facilities like safety planning. Hawes et al. (2025) accurately noted our intervention was an individually delivered treatment protocol and specifically called for research on the impact of group treatments given their dominance in the inpatient clinical setting. There are a number of features of BCBT-I that are particularly relevant to the discussion of using EBTs in inpatient settings. As the RCT demonstrated, a potentially valuable option for inpatient treatment facilities are problem-specific and strategic treatment add-ons adapted for the unique demands and limits of the inpatient setting. Although discussion of strategic treatment add-ons is common among physicians in the use of medications for the treatment of problems like refractory depression, they are rare in the psychotherapy literature, with adjunctive therapies being the more common conceptual framework.

Differentiating adjunctive therapies and strategic treatment add-ons is important. *The APA Dictionary of Psychology* (2007) defines adjunctive therapy as ‘one or more secondary interventions used concurrently with a primary intervention to enhance treatment effectiveness’, adding that it is ‘typically conducted by a different practitioner than is the primary intervention, which distinguishes it from combination therapy’. Our recent adaptation of BCBT for inpatient suicide prevention care demonstrates some of the unique characteristics of strategic treatment add-ons relative to adjunctive therapies. First, the treatment protocol was problem-specific, in this case targeting elevated suicide risk, a problem which often co-occurs with a range of Axis I and II diagnoses (Bryan & Rudd, 2006). Although problem-specific, BCBT-I provides a conceptual model that is integrative in nature and views suicide risk as part of a general model of human emotional functioning, one that applies to the full range of problems often found to co-occur with elevated suicide risk. This is not necessarily the case when isolated suicide prevention interventions are used in inpatient settings, with safety planning being arguably the most common. Second, the protocol was a dramatic reduction from the full BCBT-SP protocol, an evidence-based approach with significant RCT support (Bryan & Rudd, 2018). BCBT-I included a total of 4.5 hours in comparison to 12 hours, a 62% reduction focusing specifically on what are believed to be the essential components of the full protocol. Despite a dramatically reduced treatment protocol, BCBT-I resulted in a comparable 60% reduction in post-treatment suicide risk as the full protocol (Rudd et al., 2015) and also reduced subsequent emergency department visits by 71% and readmissions by 75% (Diefenbach et al., 2024). Third, as a treatment add-on, BCBT-I was not intended to replace the existing treatment as usual protocol; rather, it was developed to target a specific clinical problem with an evidence-based treatment. Fourth, as a treatment add-on, BCBT-I offers a flexible conceptual model that is easily integrated into the theoretical foundation of treatment as usual typical of most clinical settings. In short, when someone receiving BCBT-I attends an inpatient group session, they are unlikely to be confronted with information that conflicts with the BCBT-I conceptualization. And finally, as a strategic treatment add-on, BCBT-I was delivered individually in a manner that complemented and did not disrupt treatment as usual in the inpatient setting. As Hawes et al. (2025) mentioned, time demands during an inpatient stay are considerable. Treatment add-ons need to be developed in a way that they can accommodate the unique scheduling demands of inpatient care, with sessions scheduled in a flexible fashion.

Our experience with BCBT-I as a strategic treatment add-on for inpatient suicide prevention care has particular relevance to the discussion offered by Hawes et al. (2025) and offers a few takeaways. First, strategic treatment add-ons have distinctive and identifiable characteristics as adaptations of EBTs and are distinctly different from the traditional approach to adjunctive therapies. Second, brief treatment add-ons adapted from EBTs offer potentially promising alternatives for inpatient treatment for a wide array of problems precipitating admission in inpatient facilities and are not simply specific to elevated suicide risk. As our experience demonstrates, a problem-specific treatment add-on can be adapted to the unique demands of an inpatient setting without loss of effectiveness. Third, treatment add-ons do not necessarily need to be delivered individually but can be delivered in small groups of individuals experiencing the identified problem area. Small group delivery is an area that undeniably warrants further study and focus. And finally, strategic treatment add-ons offer a potentially unique approach to the inpatient treatment continuum that can help tailor treatment to the individual presenting problem despite both the extremely brief nature of care and the use of group therapy as the dominant modality. It is an alternative that holds considerable promise for enhancing long-term success after the transition to less intensive, outpatient treatment alternatives.
